# Supplementary material for: Multi-trait association analysis reveals shared genetic loci between Alzheimer’s disease and cardiovascular traits
Source: Nat Commun. 2024 Nov 13;15:9827. doi: 10.1038/s41467-024-53452-6 (PMC11561119; doi:10.1038/s41467-024-53452-6)
Supplement: Supplementary file 1 — Supplementary Information [file 41467_2024_53452_MOESM1_ESM.pdf]

Multi-trait association analysis reveals shared genetic loci between Alzheimer's disease and cardiovascular traits

Supplementary Figures

Supplementary Figure 1 Manhattan plots of MTAG results for A) Alzheimer's disease and B) atrial fibrillation.

Supplementary Figure 2 Manhattan plots of MTAG results for A) Alzheimer's disease and B) coronary artery disease.

Supplementary Figure 3 Manhattan plots of MTAG results for A) Alzheimer's disease and B) carotid intima-media thickness.

Supplementary Figure 4 Manhattan plots of MTAG results for A) Alzheimer's disease and B) stroke.

Supplementary Figure 5 Manhattan plots of MTAG results for A) Alzheimer's disease, B) systolic blood pressure and C) diastolic blood pressure.

Supplementary Figure 6 QQ-plots of bivariate MTAG analyses for Alzheimer's disease and the examined cardiovascular traits

Supplementary Figure 7 Regional plots of *PLEC* region across different genome-wide association studies on Alzheimer's disease

Supplementary Figure 8 Regional plots on the colocalized locı between Alzheimer's disease (bottom), cardiovascular trait (middle) and the expression quantitative trait loci for the respective tissues (top)

Supplementary Figure 9 Enriched pathways of the *PLEC*-containing module in cardiomyocytes using single cell data of cardiac tissue from heart failure cases and healthy controls.

Supplementary Figure 10 Enriched pathways of the *C1Q*-containing module in macrophages using single-nuclei transcriptomes of cardiac tissue from dilated cardiomyopathy cases and healthy controls.

Supplementary Figure 11 Enrichment of pathway genes in the protein interactomes of candidate genes in cardiomyocytes using single cell data enriched for cardiomyocytes from heart failure cases and healthy controls. The PLEC-interacting module is the turquoise and the C1Q-interacting module is the yellow.

Supplementary Figure 12 Enrichment of pathway genes in the protein interactomes of candidate genes in macrophages in cardiac tissue using single nuclei data from dilated cardiomyopathy cases and healthy controls. The PLEC-interacting module is the blue and the C1Q-interacting module is the green.

Supplementary Figure 13 Enriched pathways of the *C1Q*-containing module in microglia using single-nuclei transcriptomes of human post-mortem brain tissue from Alzheimer's disease cases and healthy controls.

Supplementary Figure 14 Pseudo-time figure of spline functions fitted to normalised gene expression for *C1Q* genes (A, *C1QA*; B, *C1QB*; C, *C1QC*) as a function of local tissue beta-amyloid load assessed as Immunohistochemical 4G8+ area in neocortical tissue from donor brains without (turquoise) or with (orange) AD. The corresponding raw data and best fit curves are shown below each panel.

Supplementary Figure 15 Enrichment of pathway genes in the protein interactomes of candidate genes in microglia using single nuclei transcriptomes of human post-mortem brain tissue from Alzheimer’s disease cases and healthy controls. The C1Q-interacting module is the turquoise.

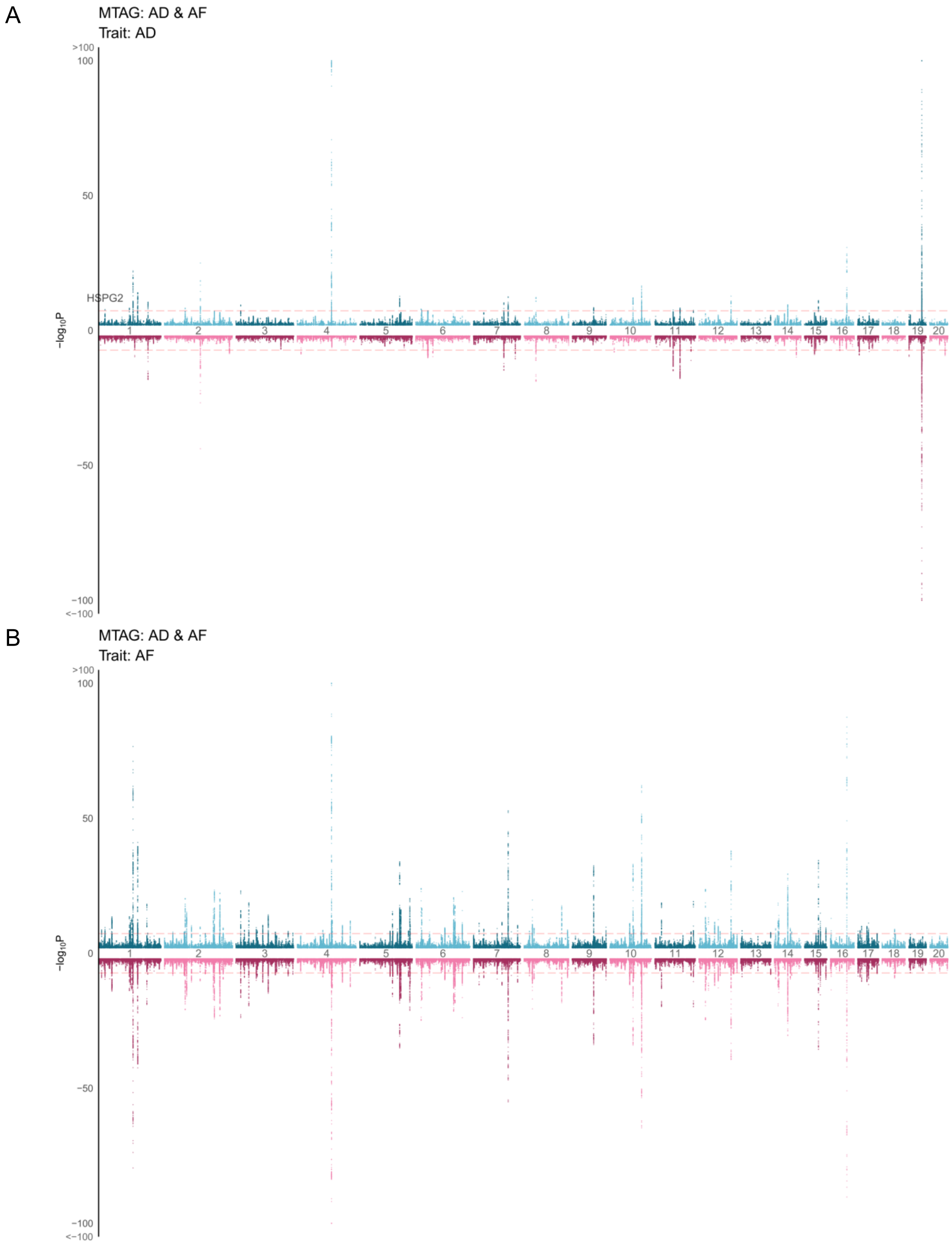

Supplementary Figure 1 Manhattan plots of MTAG results for A) Alzheimer's disease and B) atrial fibrillation.

Each mirror Manhattan plot illustrates the results from the MTAG analysis (upper part in turquoise) compared with the original GWAS results for the same trait and the same set of SNPs (lower part in red). Annotated genes denote novel discoveries identified by the MTAG analysis.
AD Alzheimer's disease, AF atrial fibrillation.

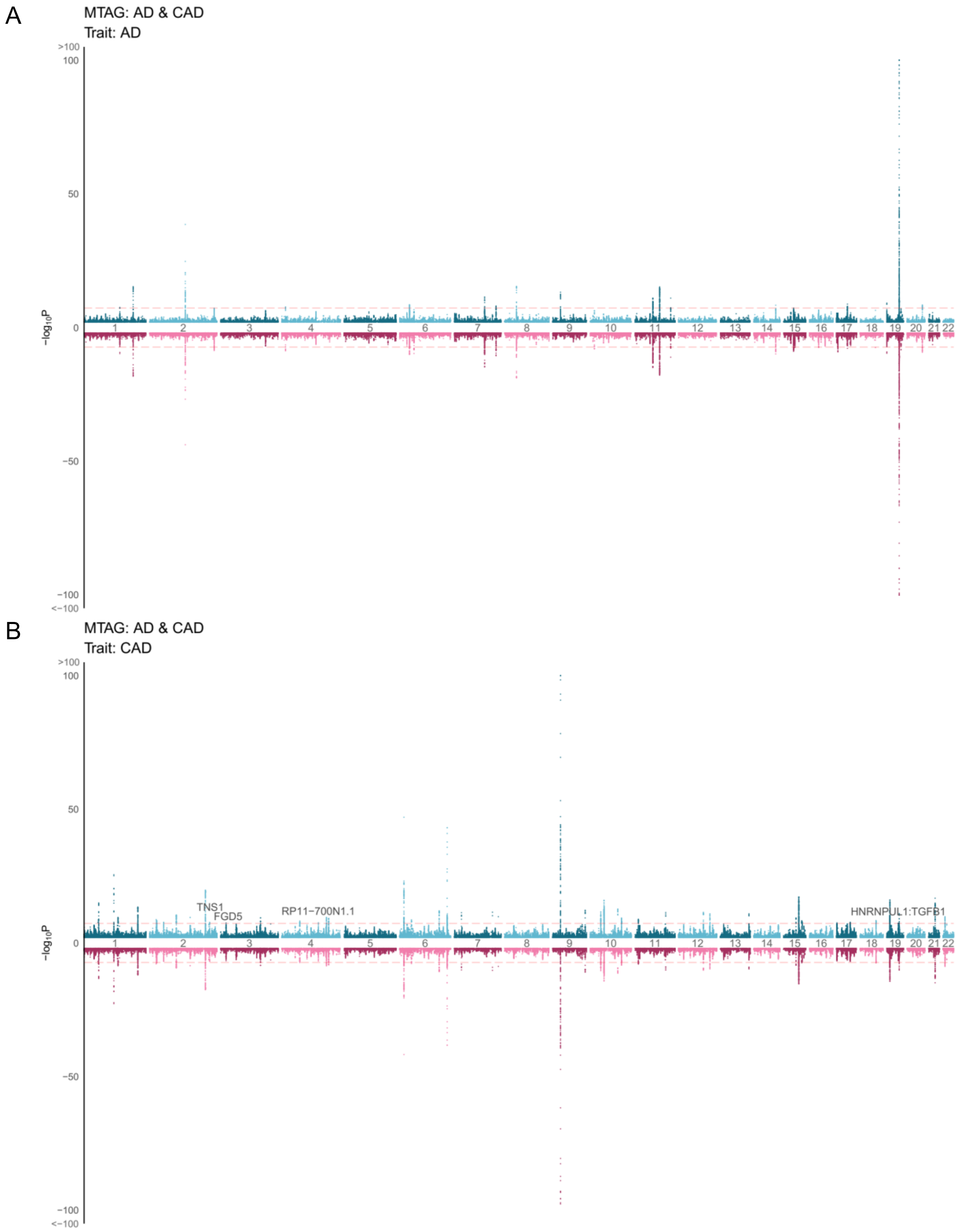

Supplementary Figure 2 Manhattan plots of MTAG results for A) Alzheimer's disease and B) coronary artery disease.

Each mirror Manhattan plot illustrates the results from the MTAG analysis (upper part in turquoise) compared with the original GWAS results for the same trait and the same set of SNPs (lower part in red). Annotated genes represent novel discoveries identified by the MTAG analysis.
AD Alzheimer's disease, CAD coronary artery disease.

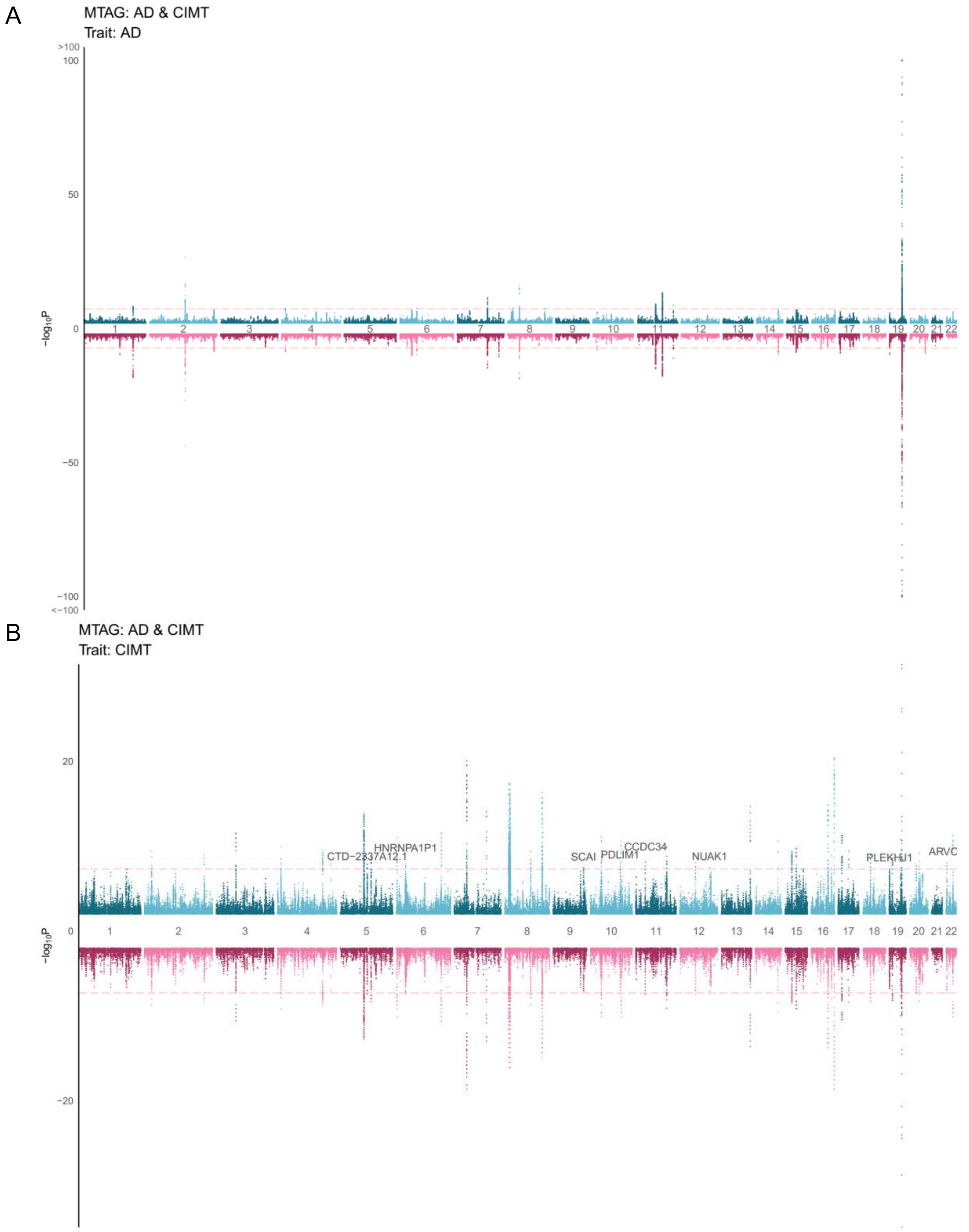

Supplementary Figure 3 Manhattan plots of MTAG results for A) Alzheimer's disease and B) carotid intima-media thickness.

Each mirror Manhattan plot illustrates the results from the MTAG analysis (upper part in turquoise) compared with the original GWAS results for the same trait and the same set of SNPs (lower part in red). Annotated genes represent novel discoveries identified by the MTAG analysis.
AD Alzheimer's disease, CIMT carotid intima-media thickness.

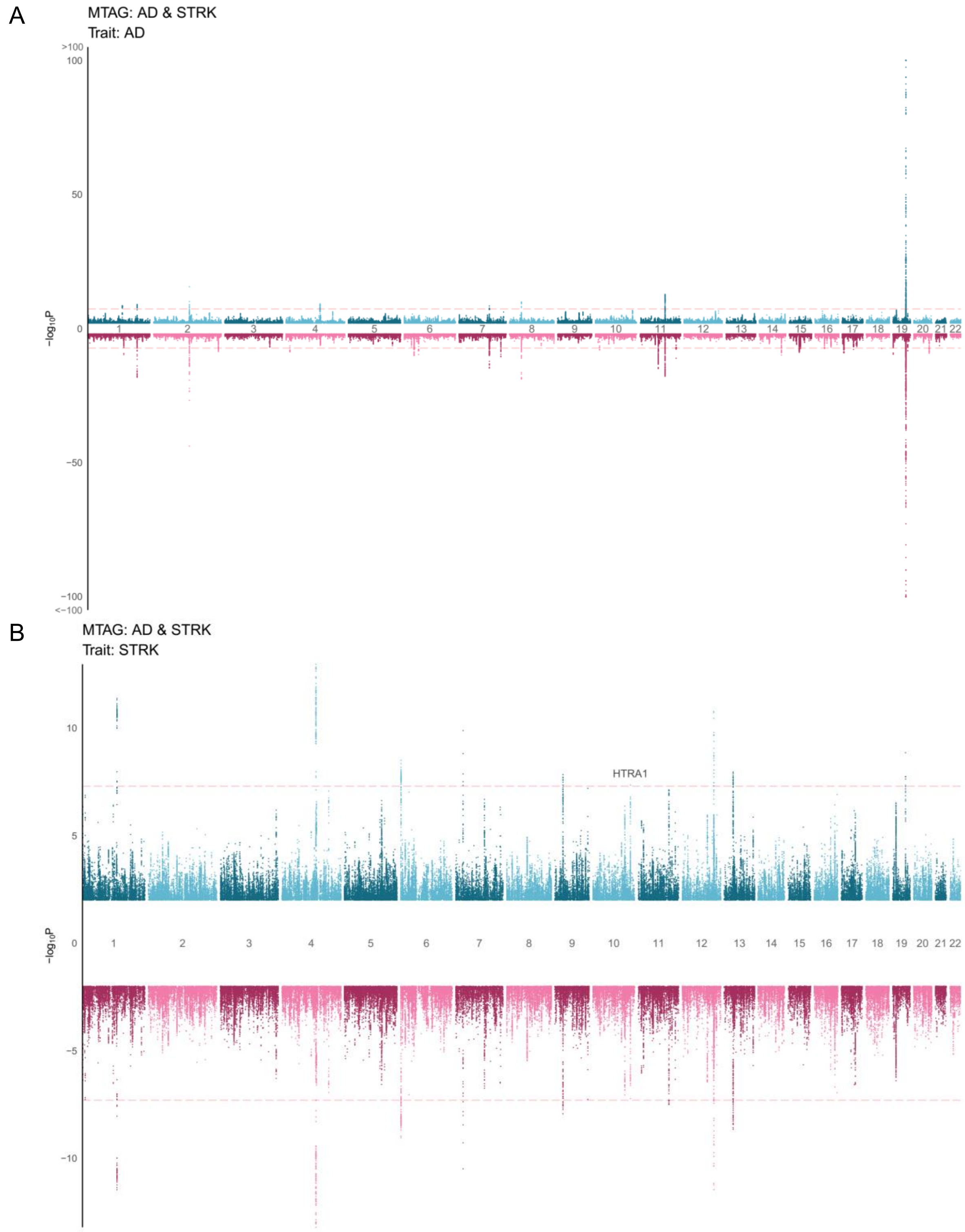

Supplementary Figure 4 Manhattan plots of MTAG results for A) Alzheimer's disease and B) stroke.

Each mirror Manhattan plot illustrates the results from the MTAG analysis (upper part in turquoise) compared with the original GWAS results for the same trait and the same set of SNPs (lower part in red). Annotated genes represent novel discoveries identified by the MTAG analysis.
AD Alzheimer's disease, STRK stroke.

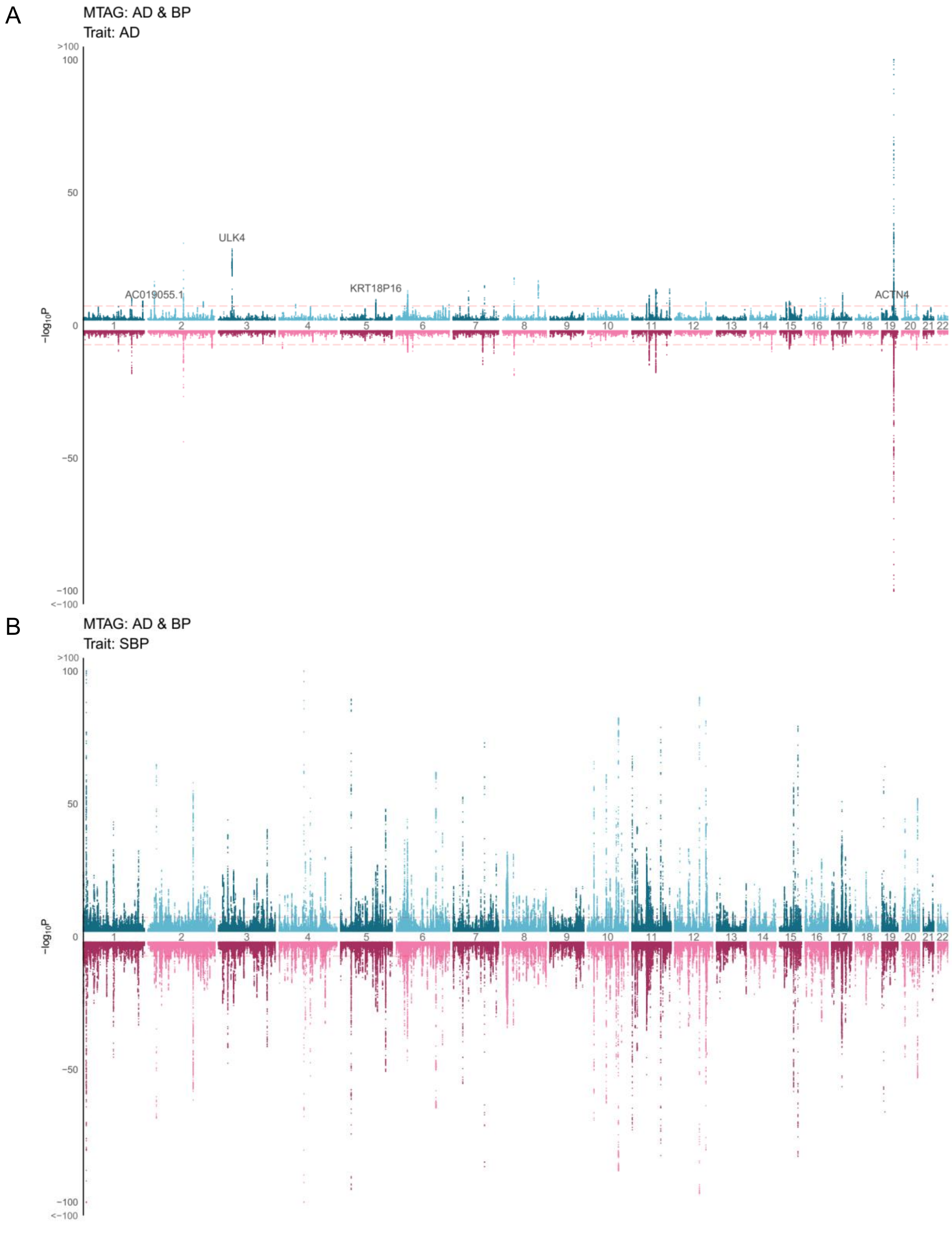
A
MTAG: AD & BP
Trait: AD
ULK4
AC019055.1
KRT18P16
ACTN4
$-log_{10}P$
>100
100
50
0
-50
-100
<-100
1 2 3 4 5 6 7 8 9 10 11 12 13 14 15 16 17 18 19 20 21 22
B
MTAG: AD & BP
Trait: SBP
$-log_{10}P$
>100
100
50
0
-50
-100
<-100
1 2 3 4 5 6 7 8 9 10 11 12 13 14 15 16 17 18 19 20 21 22

C

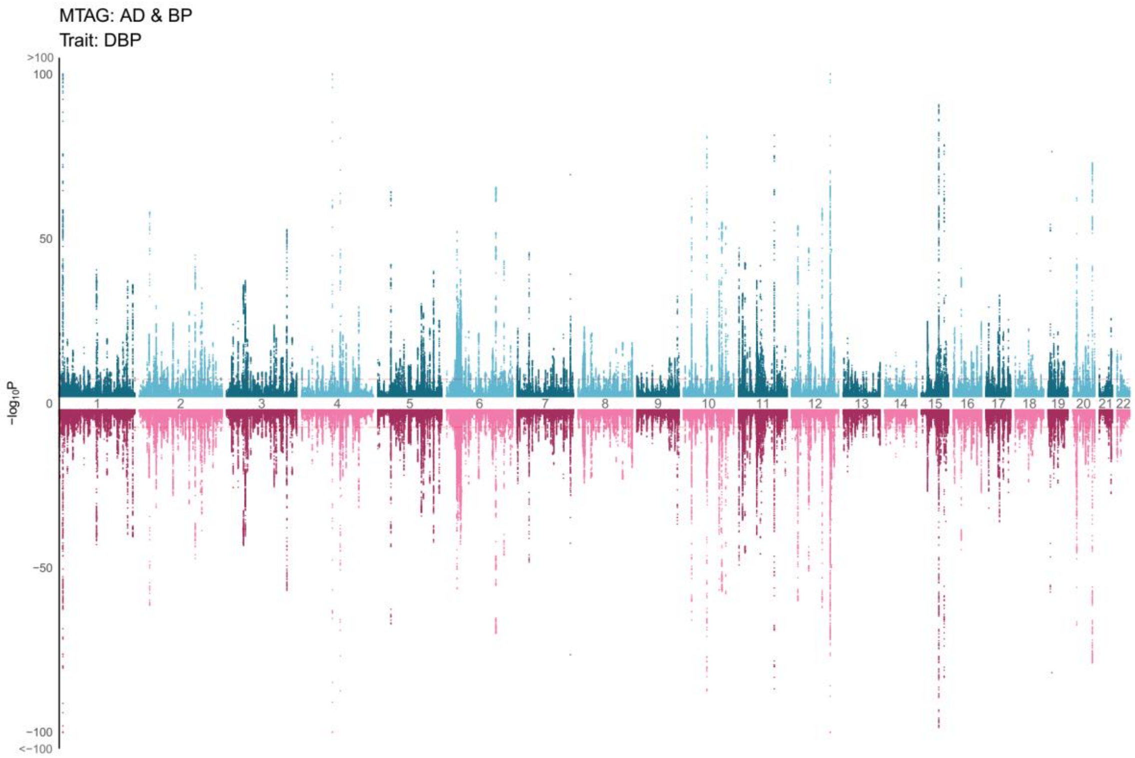

Supplementary Figure 5 Manhattan plots of MTAG results for A) Alzheimer's disease, B) systolic blood pressure and C) diastolic blood pressure.

Each mirror Manhattan plot illustrates the results from the MTAG analysis (upper part in turquoise) compared with the original GWAS results for the same trait and the same set of SNPs (lower part in red). Annotated genes represent novel discoveries identified by the MTAG analysis.
AD Alzheimer's disease, BP blood pressure, SBP systolic blood pressure, DBP diastolic blood pressure.

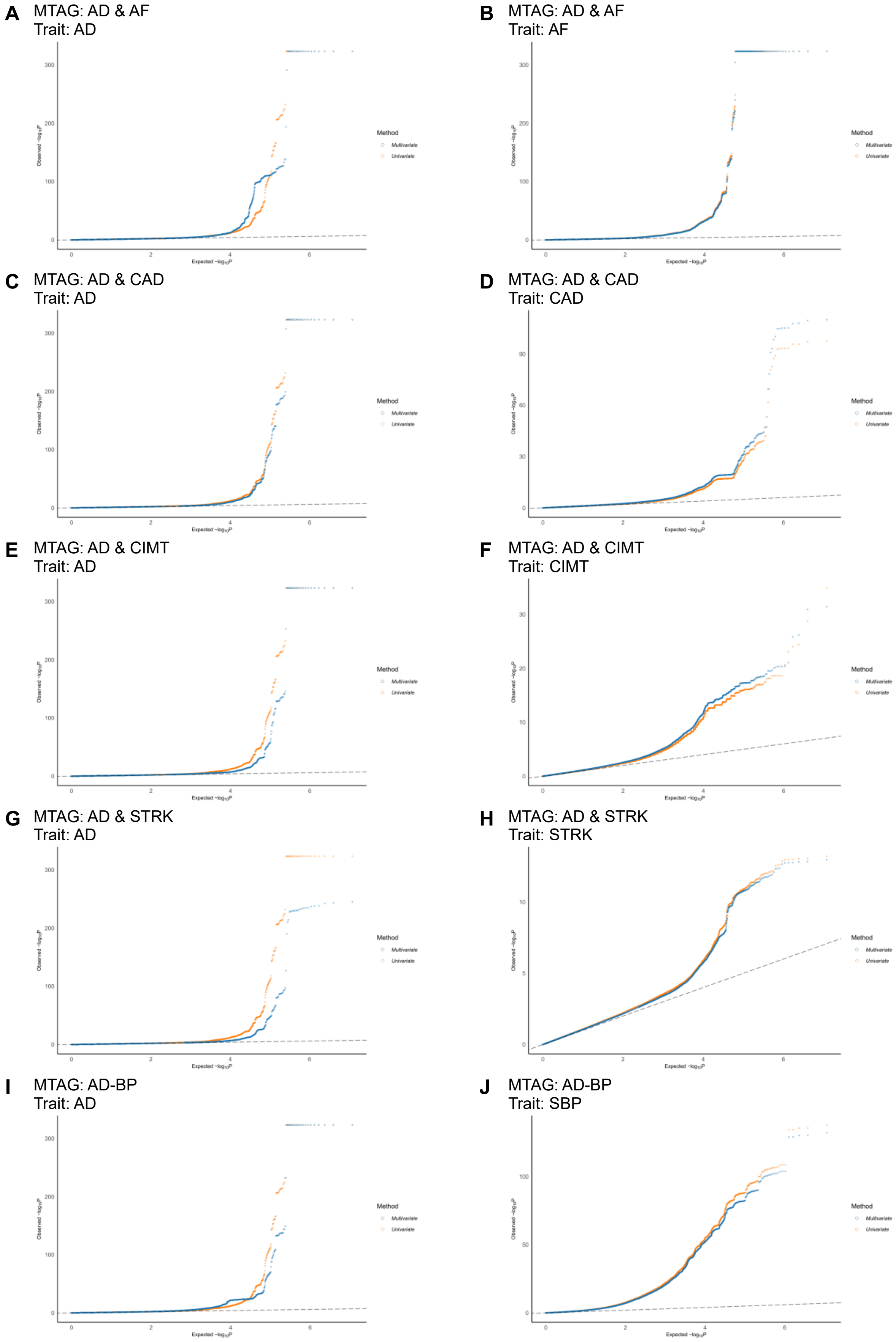
A MTAG: AD & AF
Trait: AD
B MTAG: AD & AF
Trait: AF
C MTAG: AD & CAD
Trait: AD
D MTAG: AD & CAD
Trait: CAD
E MTAG: AD & CIMT
Trait: AD
F MTAG: AD & CIMT
Trait: CIMT
G MTAG: AD & STRK
Trait: AD
H MTAG: AD & STRK
Trait: STRK
I MTAG: AD-BP
Trait: AD
J MTAG: AD-BP
Trait: SBP
Observed $-\log_{10}P$
Expected $-\log_{10}P$
Method
Multivariate
Univariate

K MTAG: AD-BP

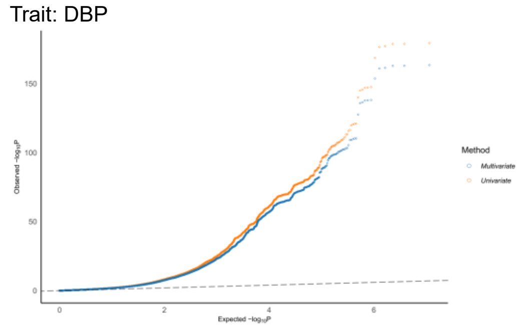

Supplementary Figure 6 QQ-plots of bivariate MTAG analyses for Alzheimer's disease and the examined cardiovascular traits

This figure presents the QQ-plots resulting from five bivariate MTAG analyses, each comparing Alzheimer's disease (AD) with a different cardiovascular trait. The examined traits alongside AD include atrial fibrillation (AF), coronary artery disease (CAD), carotid intima-media thickness (cIMT), stroke (STRK), and blood pressure (BP). Each QQ-plot displays the observed versus expected -log10(P-values) for genetic associations, comparing the MTAG results (blue) with the original GWAS results (orange) for the same trait and the same set of SNPs. The plots are organised as follows: A) AD results from the AD-AF MTAG analysis, B) AF results from the AD-AF MTAG analysis, C) AD results from the AD-CAD MTAG analysis, D) CAD results from the AD-CAD MTAG analysis, E) AD results from the AD-CIMT MTAG analysis, F) CIMT results from the AD-CIMT MTAG analysis, G) AD results from the AD-STRK MTAG analysis, H) STRK results from the AD-STRK MTAG analysis, I) AD results from the AD-BP MTAG analysis, J) SBP results from the AD-BP MTAG analysis, K) DBP results from the AD-BP MTAG analysis.

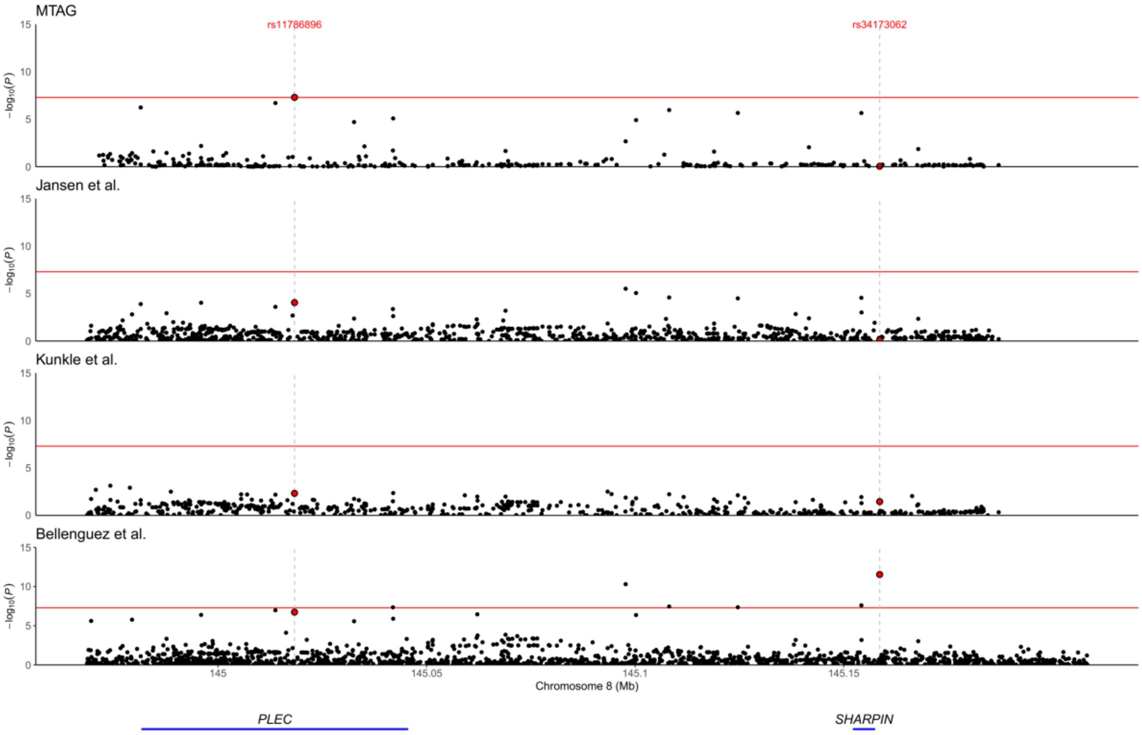

Supplementary Figure 7 Regional plots of *PLEC* region across different genome-wide association studies on Alzheimer's disease

The variant rs11786896 (*PLEC*), which was indicated by MTAG as a top signal associated with Alzheimer's disease is located approximately 150kb upstream from another previously identified variant (rs34173062). The two variants likely represent two independent signals (linkage disequilibrium r^2 = 0.006)

rs7529220

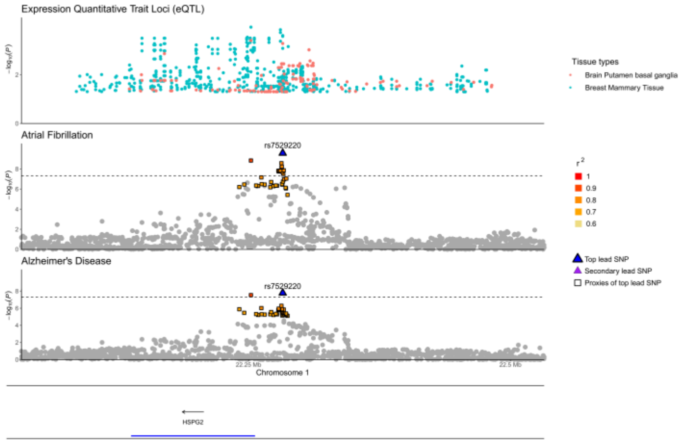

rs17362588

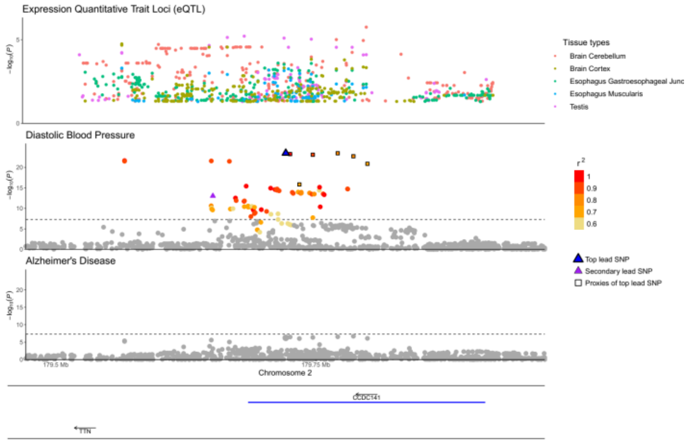

rs6738011

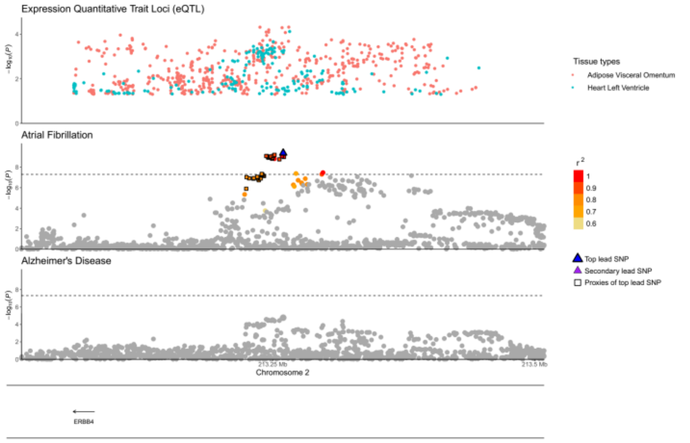

rs77399788

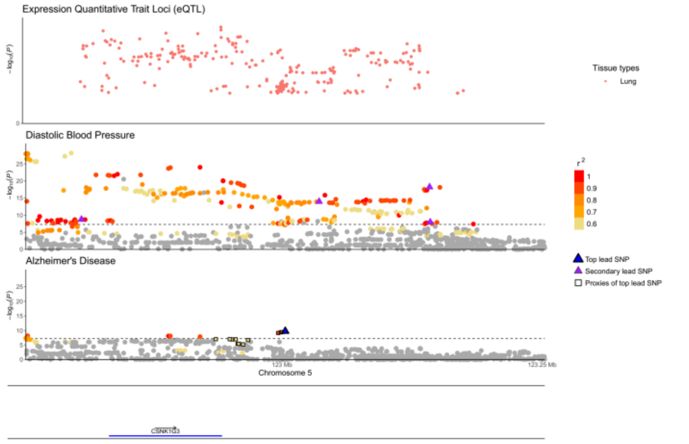

rs10949499

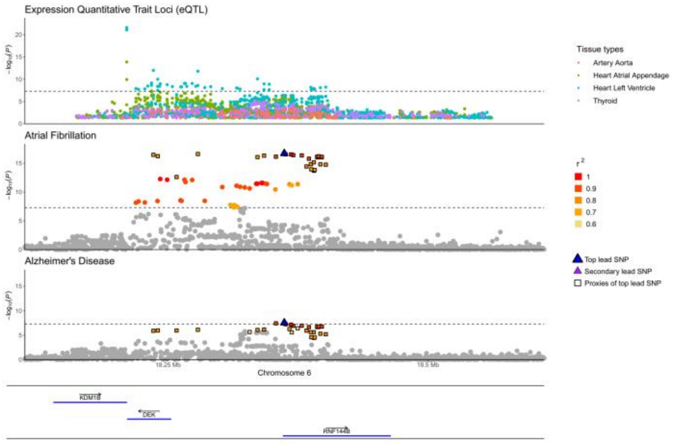

rs9399136

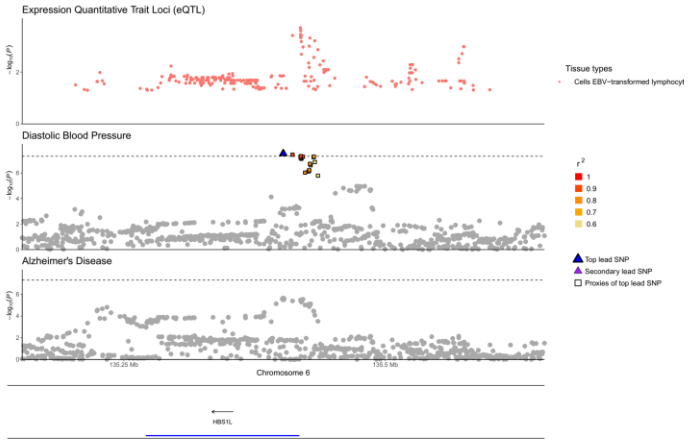

rs7834729

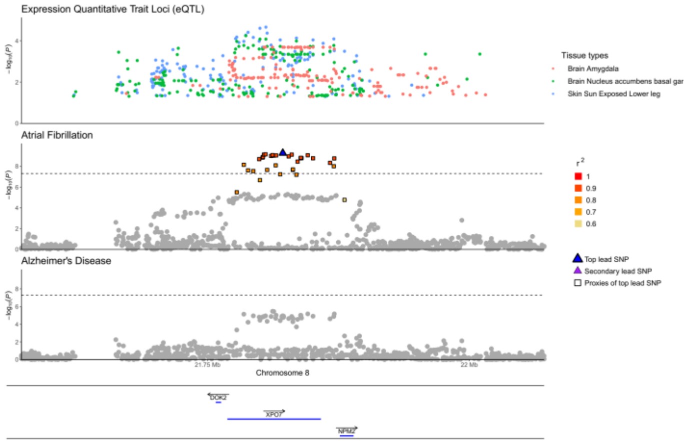

rs62521286

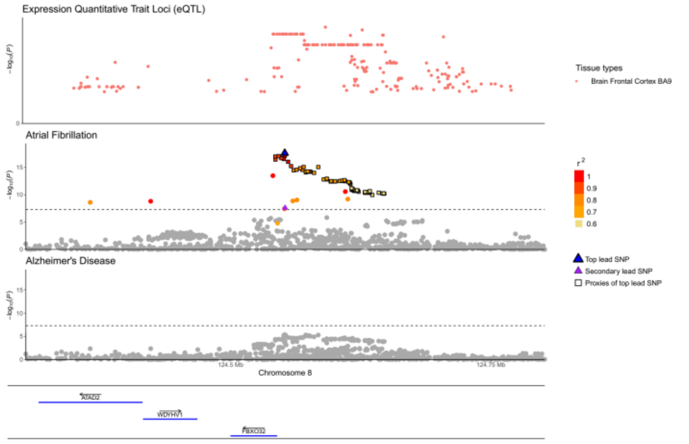

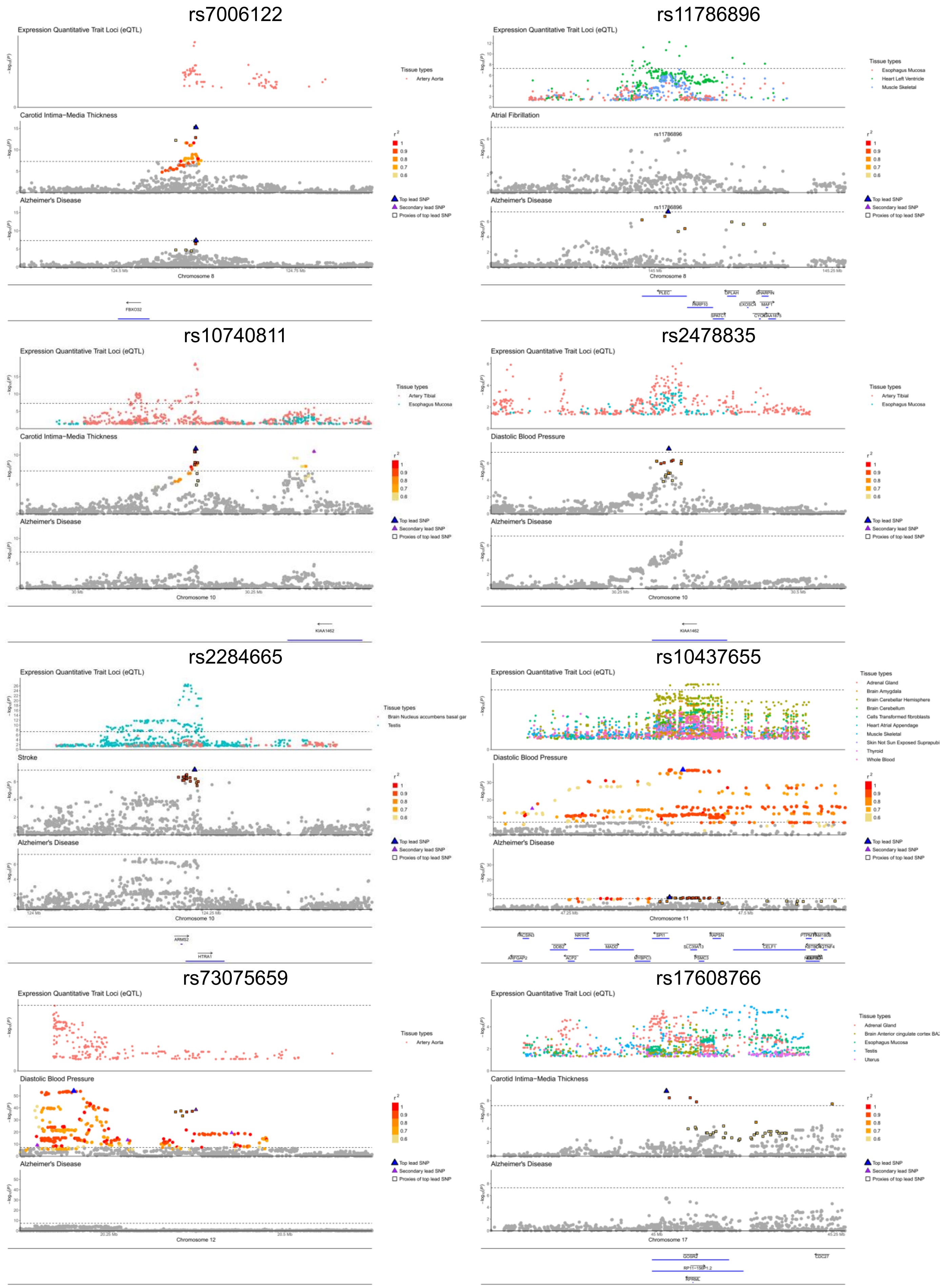

Supplementary Figure 8 Regional plots on the colocalized locı between Alzheimer's disease (bottom), cardiovascular trait (middle) and the expression quantitative trait loci for the respective tissues (top)

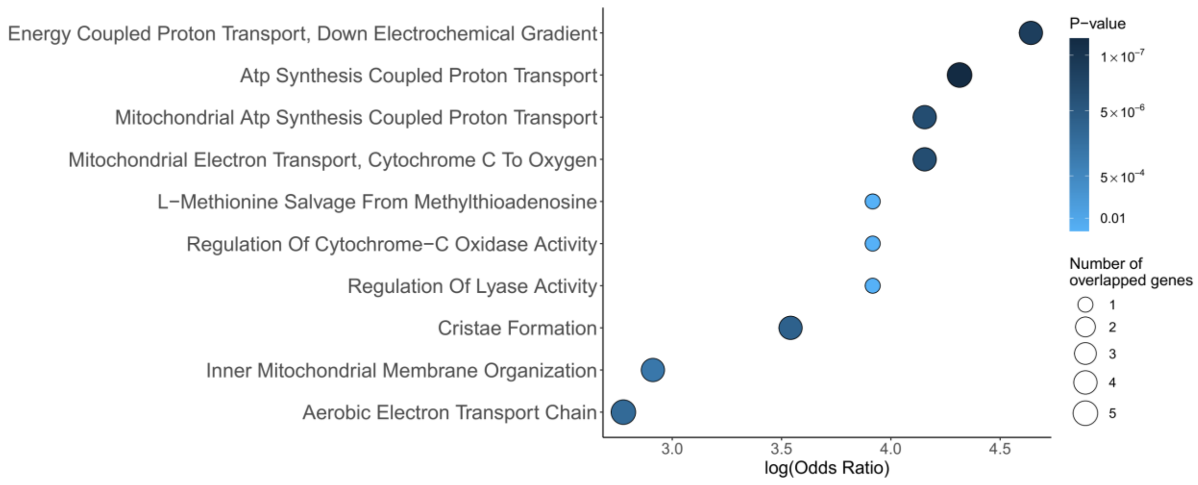

Supplementary Figure 9 **Enriched pathways of the *PLEC*-containing module in cardiomyocytes using single cell data of cardiac tissue from heart failure cases and healthy controls.**

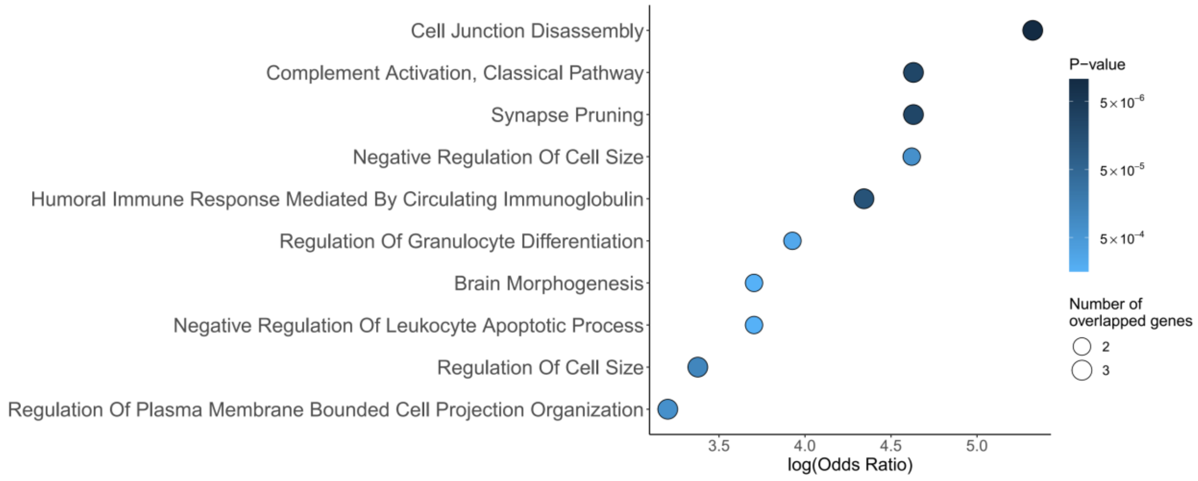

Supplementary Figure 10 **Enriched pathways of the *C1Q*-containing module in macrophages using single-nuclei transcriptomes of cardiac tissue from dilated cardiomyopathy cases and healthy controls.**

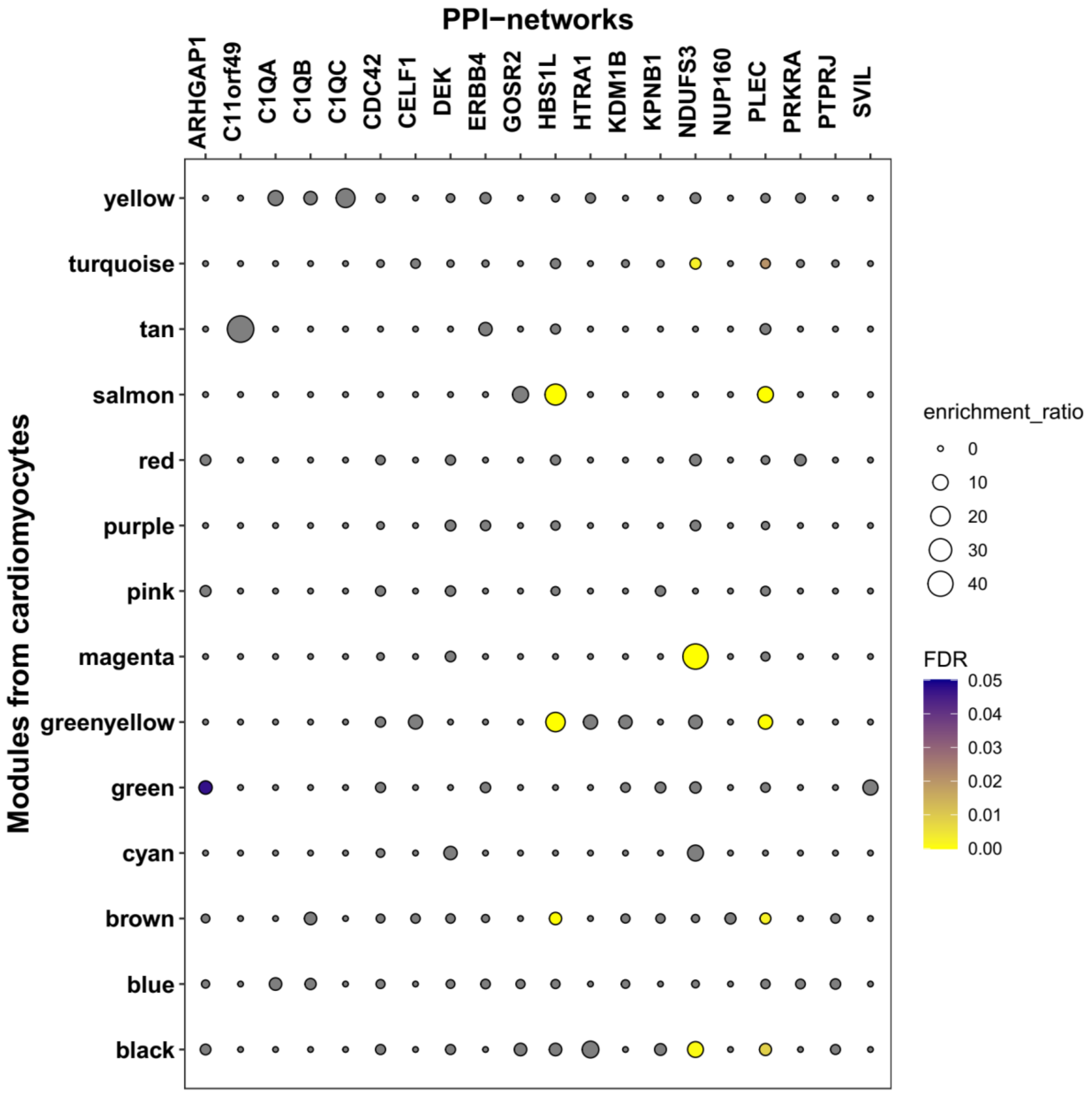

Supplementary Figure 11 Enrichment of pathway genes in the protein interactomes of candidate genes in cardiomyocytes using single cell data enriched for cardiomyocytes from heart failure cases and healthy controls. The PLEC-interacting module is the turquoise and the C1Q-interacting module is the yellow.

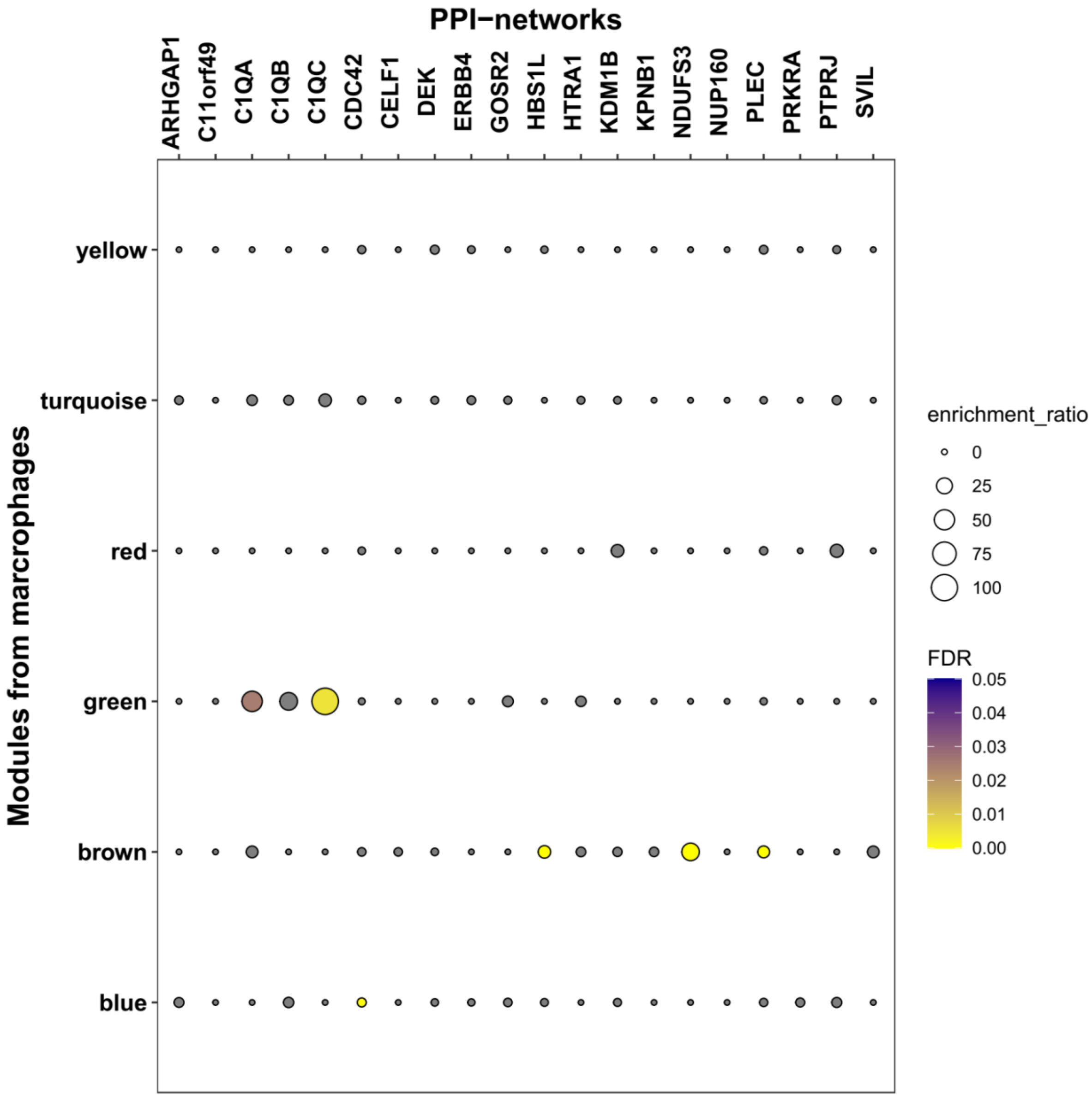

Supplementary Figure 12 **Enrichment of pathway genes in the protein interactomes of candidate genes in macrophages in cardiac tissue using single nuclei data from dilated cardiomyopathy cases and healthy controls. The PLEC-interacting module is the blue and the C1Q-interacting module is the green.**

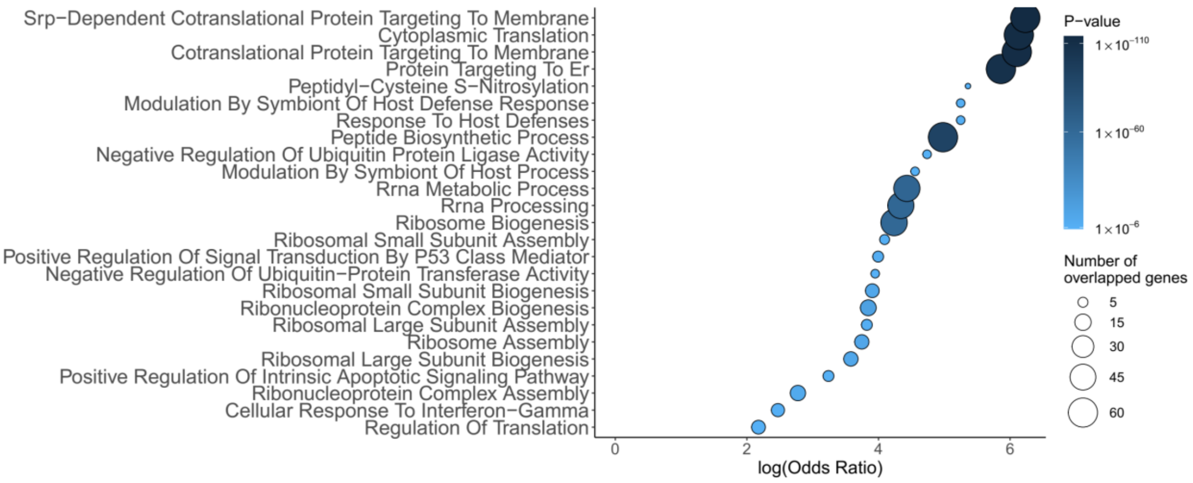

Supplementary Figure 13 Enriched pathways of the *C1Q*-containing module in microglia using single-nuclei transcriptomes of human post-mortem brain tissue from Alzheimer's disease cases and healthy controls.

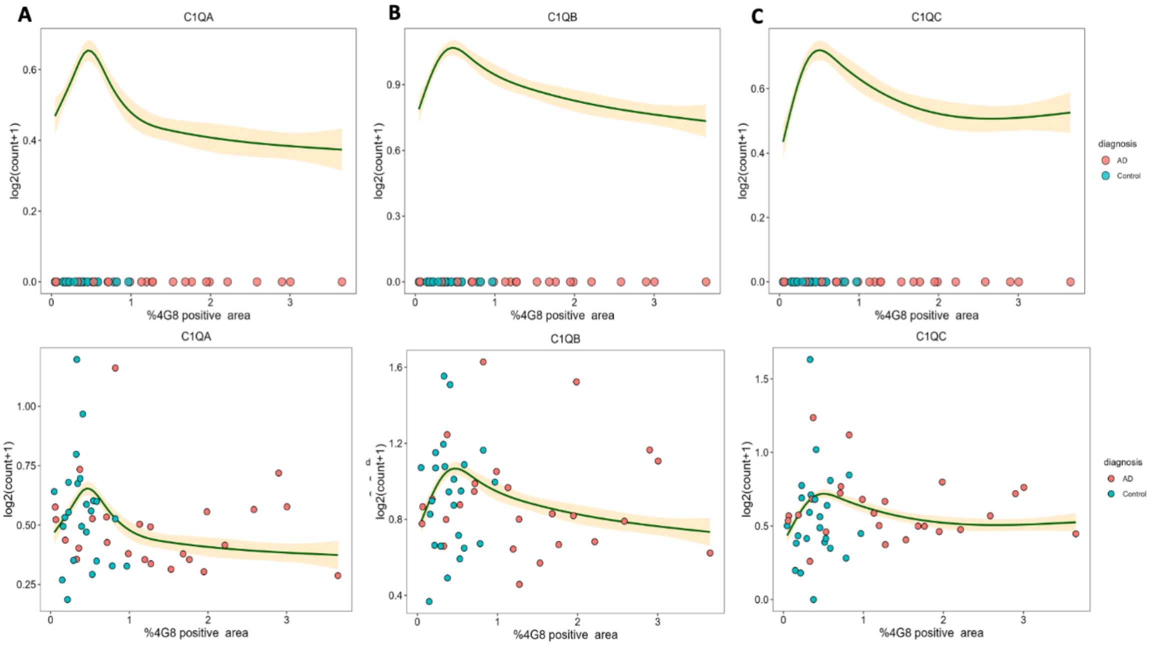

Supplementary Figure 14 Pseudo-time figure of spline functions fitted to normalised gene expression for *C1Q* genes (A, *C1QA*; B, *C1QB*; C, *C1QC*) as a function of local tissue beta-amyloid load assessed as Immunohistochemical 4G8+ area in neocortical tissue from donor brains without (turquoise) or with (orange) AD. The corresponding raw data and best fit curves are shown below each panel.

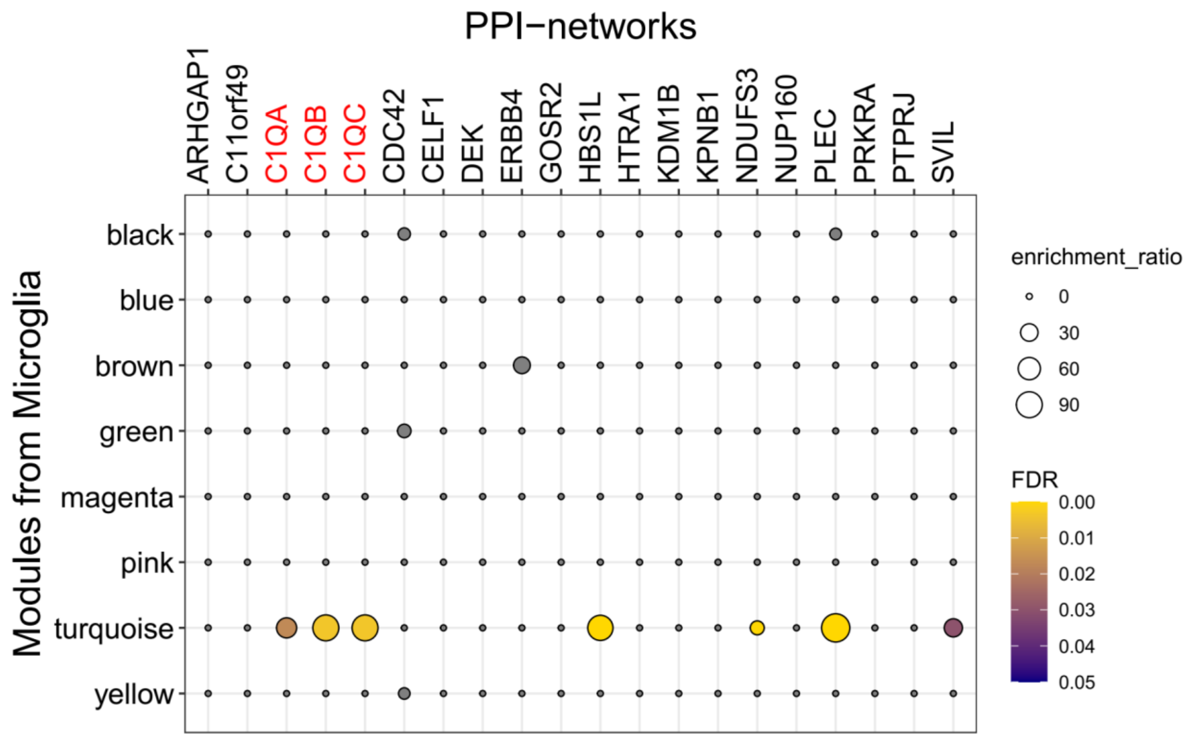

Supplementary Figure 15 Enrichment of pathway genes in the protein interactomes of candidate genes in microglia using single nuclei transcriptomes of human post-mortem brain tissue from Alzheimer's disease cases and healthy controls. The C1Q-interacting module is the turquoise.

Supplementary Tables

Supplementary Table 1 Genetic correlation between Alzheimer's disease and the examined cardiovascular traits

**Cardiovascular trait**	**r**	***P***
Atrial fibrillation	0.096	0.328
Coronary artery disease	0.046	0.653
Carotid intima-media thickness	-0.148	0.367
Stroke	0.198	0.238
Systolic blood pressure	-0.011	0.879
Diastolic blood pressure	0.050	0.513

r: Genetic correlation value; *P*: Two-sided *P*-value

Supplementary Table 2 Information of the previously published GWAS studies included in MTAG analysis

Trait	Publication	Journal	Studies	Sample Size	Source (doi)
Alzheimer's disease (AD)	Jansen et al., 2019	Nature Genetics	PGC-ALZ, ADSP, IGAP, UK Biobank	450k	10.1038/s41588-018-0311-9
Atrial fibrillation (AF)	Nielsen et al., 2018	Nature Genetics	HUNT, deCODE, MGI, DiscovEHR, UK Biobank, AFGen Consortium	1M	10.1038/s41588-018-0171-3
Coronary artery disease (CAD)	Nikpay et al., 2015	Nature Genetics	ADVANCE, AGES, ARIC, BAS, CARDIOGENICS, CAS, CCGB, COROGENE, DUKE, EGCUT, FamHS, FGENTCARD, FHS, GenRIC, GerMIFS I-IV, GoDARTS, HPS, HSDS, BioMe Biobank, ITH, LIFE-Heart, LOLIPOP, LURIC, MAYOVDB, MedStar, MIGen, OHGS, PennCATH, PIVUS, PREDICTCVD, PROCARDIS, PROMIS, PROSPER, RS, SDS/AIDHS, THISEAS, TWINGENE, ULSAM, WGHS, WTCCC	185k	10.1038/ng.3396
Carotid intima-media thickness (CIMT)	Franceschini N et al., 2018	Nature Communications	CHARGE	71k	10.1038/s41467-018-07340-5
Stroke	Malik et al., 2018	Nature Genetics	METASTROKE, NINDS-SIGN, CHARGE, EPIC, AIDHS/SDS, VHIR-FMT-Barcelona, Biobank Japan, CADISP, COMPASS, deCODE, Glasgow Stroke Sample, Helsinki 2000 Ischemic Stroke Genetics, Hisayama_FSR, HVH 1&2, INTERSTROKE, MDC, RACE, SAHLSIS, SIFAP, SLESS, UK -young lacunar stroke DNA resource, ICH	500k	10.1038/s41588-018-0058-3
Systolic blood pressure (SBP)	Evangelou et al., 2018	Nature Genetics	UK Biobank, ICBP	750k	10.1038/s41588-018-0205-x
Diastolic blood pressure (DBP)	Evangelou et al., 2018	Nature Genetics	UK Biobank, ICBP	750k	10.1038/s41588-018-0205-x

Supplementary Methods

We conducted a GWAS meta-analysis on carotid intima-media thickness (cIMT) combining data from the cohorts UK Biobank[1] and CHARGE (Cohorts for Heart and Aging Research in Genomic Epidemiology) Consortium. CHARGE GWAS has been previously described[2]. The UK Biobank (UKBB) recruited ~500,000 individuals aged 40-69 from 2006-2010 and began a pilot phase in 2014 for imaging modalities relevant to cardiovascular research, including ultrasound of the carotid arteries in 100,000 participants[3,4]. Carotid intima-media thickness (cIMT) phenotyping was performed at one of the UKBB imaging centres. A detailed description of the carotid ultrasound protocol (UKBB Resource 511; https://biobank.ctsu.ox.ac.uk/crystal/refer.cgi?id=511), as well as the genotyping measures, imputation, and central quality control performed by the UKBB, can be found elsewhere[1]. After quality control, 42,449 cIMT measurements were available for analysis. GWAS of cIMT was performed using a linear mixed non-infinitesimal model as implemented in BOLT-LMM[5], adjusted for age, sex, genotyping centre and first four principal components.

Supplementary References

1 Bycroft, C. *et al.* The UK Biobank resource with deep phenotyping and genomic data. *Nature* **562**, 203-209 (2018). https://doi.org/10.1038/s41586-018-0579-z

2 Franceschini, N. *et al.* GWAS and colocalization analyses implicate carotid intima-media thickness and carotid plaque loci in cardiovascular outcomes. *Nat Commun* **9**, 5141 (2018). https://doi.org/10.1038/s41467-018-07340-5

3 Littlejohns, T. J., Sudlow, C., Allen, N. E. & Collins, R. UK Biobank: opportunities for cardiovascular research. *Eur Heart J* **40**, 1158-1166 (2019). https://doi.org/10.1093/eurheartj/ehx254

4 Coffey, S. *et al.* Protocol and quality assurance for carotid imaging in 100,000 participants of UK Biobank: development and assessment. *Eur J Prev Cardiol* **24**, 1799-1806 (2017). https://doi.org/10.1177/2047487317732273

5 Loh, P. R., Kichaev, G., Gazal, S., Schoech, A. P. & Price, A. L. Mixed-model association for biobank-scale datasets. *Nat Genet* **50**, 906-908 (2018). https://doi.org/10.1038/s41588-018-0144-6
