# Supplementary material for: Multi-trait association analysis reveals shared genetic loci between Alzheimer’s disease and cardiovascular traits
Source: Nat Commun. 2024 Nov 13;15:9827. doi: 10.1038/s41467-024-53452-6 (PMC11561119; doi:10.1038/s41467-024-53452-6)
Supplement: Supplementary file 2 — Description of Additional Supplementary Files [file 41467_2024_53452_MOESM2_ESM.pdf]

Description of Additional Supplementary Files

File Name: Supplementary Data 1

Description: Independent signals of Alzheimer's disease (217 associations of 114 unique SNPs) indicated by five distinct MTAG analyses

File Name: Supplementary Data 2

Description: Independent signals for all examined cardiovascular traits indicated by MTAG

File Name: Supplementary Data 3

Description: Examination of the 114 unique SNPs (217 associations) associated with Alzheimer's disease across all cardiovascular traits according to MTAG results

File Name: Supplementary Data 4

Description: Genetic loci that colocalize (PP > 0.5) between Alzheimer's disease and any of the other examined traits of cardiovascular system identified by trait-trait colocalization analysis

File Name: Supplementary Data 5 Description: Genetic loci that colocalize (PP > 0.5) between Alzheimer's disease and traits of the cardiovascular system via a shared gene that is expressed in at least one of the examined tissues identified by trait-exression quantitative trait loci (eQTL) colocalization analysis

File Name: Supplementary Data 6

Description: List of genes per module for each examined cell type from cardiovascular tissue constructed by WGCNA

File Name: Supplementary Data 7

Description: Differential module eigengenes analysis between heart failure cases and healthy controls in cardiac vascular endothelial cells and cardiomyocytes and between

dilated cardiomyopathy cases and healthy controls in macrophages

File Name: Supplementary Data 8

Description: Pathways enriched for PLEC-containing modules between heart failure cases and healthy controls in cardiac vascular endothelial cells and cardiomyocytes and between dilated cardiomyopathy cases and healthy controls in macrophages

File Name: Supplementary Data 9

Description: Pathways enriched for C1q-containing modules between heart failure cases and healthy controls in cardiac vascular endothelial cells and cardiomyocytes and between dilated cardiomyopathy cases and healthy controls in macrophages

File Name: Supplementary Data 10

Description: List of genes per interactome used in protein-protein interaction analysis

File Name: Supplementary Data 11

Description: Protein-protein interaction analysis for PLEC, NDUFS3 and C1Q interactomes across cell types of human left ventricular tissue

File Name: Supplementary Data 12

Description: List of genes per module for each examined cell type from brain tissue constructed by WGCNA

File Name: Supplementary Data 13

Description: Differential module eigengenes analysis between Alzheimer's disease cases and healthy controls in astrocytes and microglia

File Name: Supplementary Data 14

Description: Pathways enriched for PLEC-containing and C1Q- containing modules between

Alzheimer's disease cases and healthy controls in brain astrocytes and microglia

File Name: Supplementary Data 15

Description: Pathways enriched for PLEC-interacting and NDUFS3-interacting modules in microglia between Alzheimer's disease cases and healthy controls

File Name: Supplementary Data 16

Description: Protein-protein interaction analysis for PLEC and NDUFS3 interactomes in brain astrocytes and C1Q interactome in brain microglia
