# Supplementary material for: Multi-trait association analysis reveals shared genetic loci between Alzheimer’s disease and cardiovascular traits
Source: Nat Commun. 2024 Nov 13;15:9827. doi: 10.1038/s41467-024-53452-6 (PMC11561119; doi:10.1038/s41467-024-53452-6)
Supplement: Supplementary file 4 — Reporting Summary [file 41467_2024_53452_MOESM4_ESM.pdf]

# nature portfolio

Corresponding author(s): Fotios Koskeridis

Last updated by author(s): Sep 25, 2024

# Reporting Summary

Nature Portfolio wishes to improve the reproducibility of the work that we publish. This form provides structure for consistency and transparency in reporting. For further information on Nature Portfolio policies, see our Editorial Policies and the Editorial Policy Checklist.

## Statistics

For all statistical analyses, confirm that the following items are present in the figure legend, table legend, main text, or Methods section.

| n/a | Confirmed | |
|---|---|---|
| ☐ | ☒ | The exact sample size (*n*) for each experimental group/condition, given as a discrete number and unit of measurement |
| ☐ | ☒ | A statement on whether measurements were taken from distinct samples or whether the same sample was measured repeatedly |
| ☐ | ☒ | The statistical test(s) used AND whether they are one- or two-sided<br>*Only common tests should be described solely by name; describe more complex techniques in the Methods section.* |
| ☐ | ☒ | A description of all covariates tested |
| ☐ | ☒ | A description of any assumptions or corrections, such as tests of normality and adjustment for multiple comparisons |
| ☐ | ☒ | A full description of the statistical parameters including central tendency (e.g. means) or other basic estimates (e.g. regression coefficient) AND variation (e.g. standard deviation) or associated estimates of uncertainty (e.g. confidence intervals) |
| ☐ | ☒ | For null hypothesis testing, the test statistic (e.g. *F*, *t*, *r*) with confidence intervals, effect sizes, degrees of freedom and *P* value noted<br>*Give P values as exact values whenever suitable.* |
| ☐ | ☒ | For Bayesian analysis, information on the choice of priors and Markov chain Monte Carlo settings |
| ☒ | ☐ | For hierarchical and complex designs, identification of the appropriate level for tests and full reporting of outcomes |
| ☐ | ☒ | Estimates of effect sizes (e.g. Cohen's *d*, Pearson's *r*), indicating how they were calculated |

*Our web collection on statistics for biologists contains articles on many of the points above.*

## Software and code

Policy information about availability of computer code

| | |
|---|---|
| Data collection | No software or code was used for the data collection in this study |
| Data analysis | MTAG v.1.0.8, R v4.4.0, FUMA v1.3.7, PLINK v1.9, HyPrColoc v1.0.0, scFlow 0.7.4, rliger 2.0.1, EWCE 1.13.1, hdWGCNA 0.3.03, STRINGdb 1.8.1 |

For manuscripts utilizing custom algorithms or software that are central to the research but not yet described in published literature, software must be made available to editors and reviewers. We strongly encourage code deposition in a community repository (e.g. GitHub). See the Nature Portfolio guidelines for submitting code & software for further information.

## Data

Policy information about availability of data

All manuscripts must include a data availability statement. This statement should provide the following information, where applicable:

- Accession codes, unique identifiers, or web links for publicly available datasets
- A description of any restrictions on data availability
- For clinical datasets or third party data, please ensure that the statement adheres to our policy

The summary statistics from the GWAS included in this study are publicly available and can be retrieved from GWAS Catalog under the accession codes GCST007320 (AD), GCST006414 (AF), GCST003116 (CAD), GCST006906 (stroke), GCST006624 (SBP) and GCST006630 (DBP). Heart single-cell data from the left ventricle tissue were downloaded from Gene Expression Omnibus (GEO) under accession codes GSE109816 (cardiomyocytes-enriched samples) and GSE121893 (normal digested samples). Single-nuclei data for the left ventricular tissue were retrieved from GEO under accession code GSE109816. The single-nuclei RNA sequencing data for human post-mortem brain samples from AD and Control samples were retrieved from GEO under accession code GSE160936. The MTAG summary statistics

generated in this study have been deposited in NHGRI-EBI GWAS Catalog under accession codes GCST90449053 (AD from AD-AF MTAG), GCST90449054 (AF), GCST90449055 (AD from AD-BP MTAG), GCST90449056 (SBP), GCST90449057 (DBP), GCST90449058 (AD from AD-CAD MTAG), GCST90449059 (CAD), GCST90449060 (AD from AD-cIMT MTAG), GCST90449061 (cIMT), GCST90449062 (AD from AD-stroke MTAG), and GCST90449063 (stroke). All other data generated in this study are provided with this published article (and its supplementary information files).

## Research involving human participants, their data, or biological material

Policy information about studies with human participants or human data. See also policy information about sex, gender (identity/presentation), and sexual orientation and race, ethnicity and racism.

| | |
|---|---|
| Reporting on sex and gender | This study used summary statistics from previously published GWAS studies including males and females. Any information regarding the sex and gender definition is described in the respective study. References to the included studies are provided in Supplementary Table 18. No sex- or gender-based analysis was performed in this study. |
| Reporting on race, ethnicity, or other socially relevant groupings | This study used summary statistics from previously published GWAS studies on population of European ancestry. The population characteristics of the included studies are described in the respective study and references to the included studies are provided in Supplementary Table 18. |
| Population characteristics | This study used summary statistics from previously published GWAS studies on population of European ancestry. The population characteristics of the included studies are described in the respective study and references to the included studies are provided in Supplementary Table 18. |
| Recruitment | This study used summary statistics from previously published GWAS studies. No additional recruitment was performed for the purposes of those analyses. |
| Ethics oversight | This study used summary statistics from previously published GWAS studies. The ethical approvals for each included GWAS study are described in the respective study and references to the included studies are provided in Supplementary Table 18. |

Note that full information on the approval of the study protocol must also be provided in the manuscript.

# Field-specific reporting

Please select the one below that is the best fit for your research. If you are not sure, read the appropriate sections before making your selection.

[x] Life sciences [ ] Behavioural & social sciences [ ] Ecological, evolutionary & environmental sciences

For a reference copy of the document with all sections, see nature.com/documents/nr-reporting-summary-flat.pdf

# Life sciences study design

All studies must disclose on these points even when the disclosure is negative.

| | |
|---|---|
| Sample size | This study used summary statistics from previously published GWAS studies. As a rule, the largest GWAS of European ancestry for the respective trait was used. The sample of the included GWAS ranged from 185,000 to 1,030,000 participants. |
| Data exclusions | All insertion and deletion polymorphisms, rare variants (MAF < 0.01), variants with a sample size less than 2/3 of the 90th percentile and palindromic SNPs were excluded from the analysis. |
| Replication | This study used summary statistics from publicly available GWAS, therefore a standard replication was not applicable. Instead, we performed a Summary-data-based Mendelian randomization (SMR) to validate our novel signals and a genetic colocalization analysis to provide additional evidence for discovered pleiotropic associations. |
| Randomization | This study used summary statistics from publicly available GWAS. Randomization was not applicable. |
| Blinding | This study used summary statistics from publicly available GWAS. Blinding was not applicable. |

# Reporting for specific materials, systems and methods

We require information from authors about some types of materials, experimental systems and methods used in many studies. Here, indicate whether each material, system or method listed is relevant to your study. If you are not sure if a list item applies to your research, read the appropriate section before selecting a response.

## Materials & experimental systems

| n/a | Involved in the study |
|---|---|
| ☒ | ☐ Antibodies |
| ☒ | ☐ Eukaryotic cell lines |
| ☒ | ☐ Palaeontology and archaeology |
| ☒ | ☐ Animals and other organisms |
| ☒ | ☐ Clinical data |
| ☒ | ☐ Dual use research of concern |
| ☒ | ☐ Plants |

## Methods

| n/a | Involved in the study |
|---|---|
| ☒ | ☐ ChIP-seq |
| ☒ | ☐ Flow cytometry |
| ☒ | ☐ MRI-based neuroimaging |

## Plants

| | |
|---|---|
| Seed stocks | *Report on the source of all seed stocks or other plant material used. If applicable, state the seed stock centre and catalogue number. If plant specimens were collected from the field, describe the collection location, date and sampling procedures.* |
| Novel plant genotypes | *Describe the methods by which all novel plant genotypes were produced. This includes those generated by transgenic approaches, gene editing, chemical/radiation-based mutagenesis and hybridization. For transgenic lines, describe the transformation method, the number of independent lines analyzed and the generation upon which experiments were performed. For gene-edited lines, describe the editor used, the endogenous sequence targeted for editing, the targeting guide RNA sequence (if applicable) and how the editor was applied.* |
| Authentication | *Describe any authentication procedures for each seed stock used or novel genotype generated. Describe any experiments used to assess the effect of a mutation and, where applicable, how potential secondary effects (e.g. second site T-DNA insertions, mosiacism, off-target gene editing) were examined.* |
